# A fast-converging iterative method based on weighted feedback for multi-distance phase retrieval

**DOI:** 10.1038/s41598-018-24666-8

**Published:** 2018-04-24

**Authors:** Cheng Guo, Cheng Shen, Qiang Li, Jiubin Tan, Shutian Liu, Xinchi Kan, Zhengjun Liu

**Affiliations:** 1 Department of Automatic test and control, Harbin Institute of Technology, Harbin, 150080 China; 2 Department of Physics, Harbin Institute of Technology, Harbin, 150080 China; 3 College of Veterinary Medicine, Jilin University, Changchun, 130062 China

## Abstract

Multiple distance phase retrieval methods hold great promise for imaging and measurement due to their less expensive and compact setup. As one of their implementations, the amplitude-phase retrieval algorithm (APR) can achieve stable and high-accuracy reconstruction. However, it suffers from the slow convergence and the stagnant issue. Here we propose an iterative modality named as weighted feedback to solve this problem. With the plug-ins of single and double feedback, two augmented approaches, i.e. the APRSF and APRDF algorithms, are demonstrated to increase the convergence speed with a factor of two and three in experiments. Furthermore, the APRDF algorithm can extend the multiple distance phase retrieval to the partially coherent illumination and enhance the imaging contrast of both amplitude and phase, which actually relaxes the light source requirement. Thus the weighted feedback enables a fast-converging and high-contrast imaging scheme for the iterative phase retrieval.

## Introduction

Phase retrieval methods^1^, which have been applied in various fields of science and engineering, including X-ray imaging^2^, quantum imaging^3^, astronomy^4^, super-resolution^5^, diffraction tomography^6^, and wide-field imaging^7^, enable one to recover the phase information of an object from the magnitude of its diffraction pattern. As a fundamental theory of the iterative phase retrieval methods, the Gerchberg–Saxton algorithm (GS)^8^ was invented to retrieve the object’s phase with a pair of intensity patterns located in the object and recording planes. The original GS algorithm was slow and sensitive to initial guesses. To solve this problem, Fienup improved the GS algorithm with a support constraint of non-negativity and a pre-assigned boundary to speed up the convergence under single or double measurements^9,10^. However, these methods both have to get a rough estimation for the object. Also, since they are defined in far field region^11–13^, it is not suitable for a compact imaging system. Compared to these single and double measurement schemes, the multi-image phase retrieval methods are investigated by virtue of measurement diversities^14–26^. Without the need of the support constraint, these methods are capable of obtaining an optimal convergence and high-accuracy reconstruction by utilizing different scanning strategies, such as overlapping illumination^15–17^, multi-wavelength scanning^18^, multi-angle illumination^19^, pinhole scanning^26^ and multiple distance measurements^14,22–25^.

Due to the compact implementation and low cost, the multiple distance phase retrieval methods attract more concerns in the imaging field, which have been applied in lens-based imaging systems^21,24^, the coherent diffractive imaging system^14^ and the digital in-line holography^22,27,28^. The strategy of multi-positioned measurements is fully compatible with all kinds of light propagation models, such as Fourier transform^5^, gyrator transform^20^ and Fresnel diffraction^15^. As one of the multiple distance phase retrievals, the amplitude-phase retrieval algorithm (APR) appropriately combines the reconstruction accuracy and stability for imaging^23,25^. However, it is heavily enslaved to the slow convergence rate. Generally, a satisfactory result of the APR algorithm is obtained at the expense of more measurements or iterations, both of which demand heavy computational loads. Moreover, the long-time computation may not guarantee an improved result due to its stagnant issue.

In this work, we propose an acceleration modality for the APR algorithm, named as the weighted feedback. The weighted feedback can effectively enhance the convergence speed. The corresponding feedback coefficients are optimized by the data-driven curve fitting. Simulations and experiments have demonstrated the acceleration validity. To further prove the stability of weighted feedback, this phase retrieval method is applied in the imaging system with partially coherent illumination. The result indicates that the weighted feedback APR is capable of obtaining an enhanced imaging contrast for different objects.

## Method

**Figure 1:**
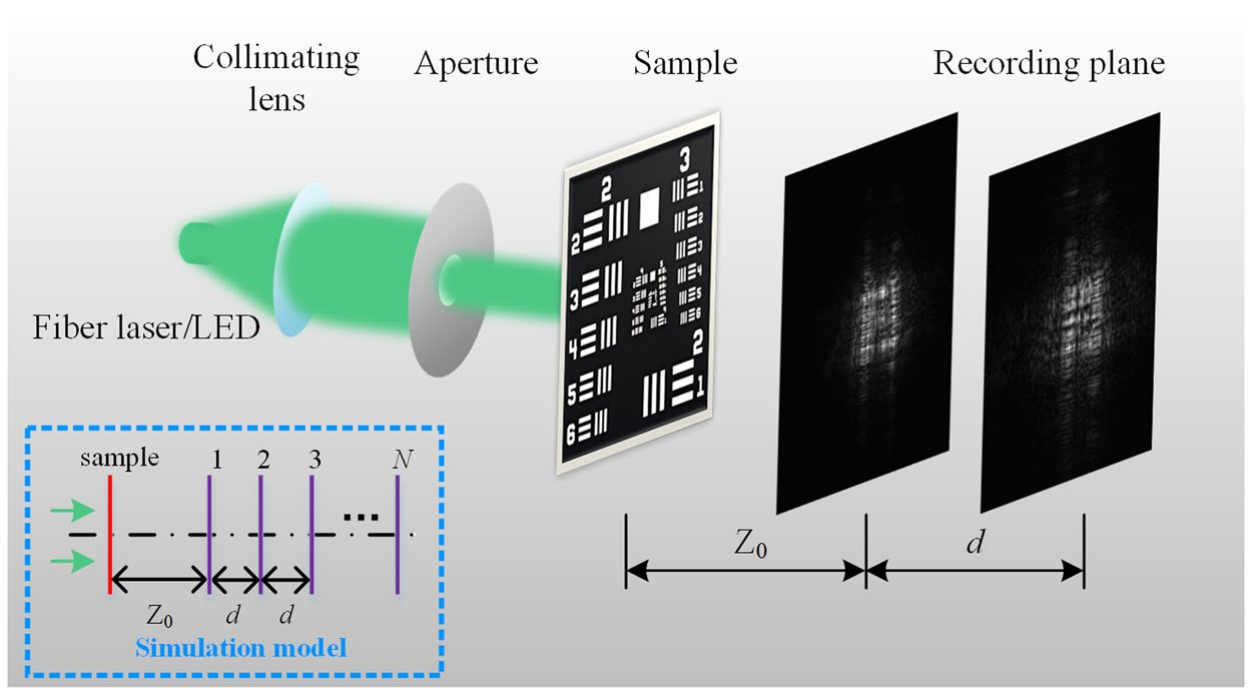
The imaging schematic of multi-distance phase retrieval.

**Figure 2:**
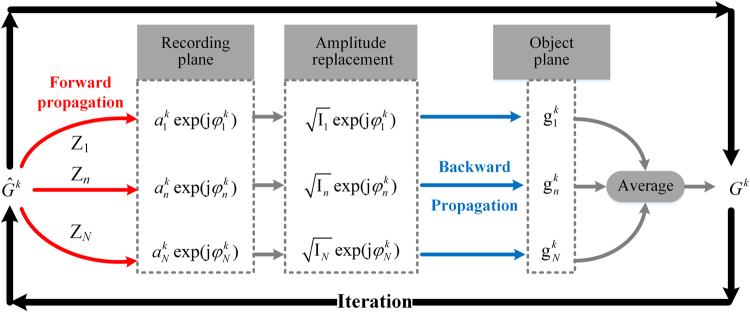
The flowchart of multi-distance phase retrieval.

We firstly describe the notation and basic concepts of the APR algorithm and the weighted feedback. The experimental schematic and flowchart of APR algorithm are given in Figs 1 and 2. As a beam of plane wave illuminates sample, a set of diffraction patterns I_*n*_(*n* ∈ [1, *N*]) measured with different transverse distances in the diffractive downstream are able to achieve complex-valued image reconstruction of the object. To simplify the notation, the transverse distances Z_*n*_ are described as Z_*n*_ = Z_0_ + (*n* − 1)*d*, which are composed of initial distance Z_0_ and equivalent interval *d*. The complex amplitude of object is reconstructed by iterative back-and-forth propagation computation. Here the computation of diffraction propagation is based on Fresnel region, whose transfer function is expressed as

1 $${H}_{n}(\xi ,\eta )=\{\begin{array}{cc}\exp [\frac{{\rm{2}}\pi {{\rm{jZ}}}_{n}}{\lambda }\sqrt{1-{(\lambda \xi )}^{2}-{(\lambda \eta )}^{2}}], & {(\lambda \xi )}^{2}+{(\lambda \eta )}^{2} < 1,\\ 0\,, & \mathrm{otherwise},\end{array}$$

where *λ* is wavelength of the illumination and (*ξ*, *η*) is the coordinate in frequency domain. Thus the forward and backward propagation of an input signal *S* related to transverse distance Z_*n*_ are accomplished by ${{\boldsymbol{ {\mathcal F} }}}^{-1}[{\boldsymbol{ {\mathcal F} }}(S){H}_{n}]$ and ${{\boldsymbol{ {\mathcal F} }}}^{-1}[{\boldsymbol{ {\mathcal F} }}(S){H}_{n}^{\ast }]$, where ${\boldsymbol{ {\mathcal F} }}$ is the Fourier transform and the superscript ‘*’ denotes the complex conjugate.

The detailed algorithmic flowchart of APR is depicted in Fig. 2: (1) initializing complex amplitude with constant matrix; (2) propagating *k-*th input of object’s complex filed ${\hat{G}}^{k}$ forward to the recording plane and then obtaining the computed diffraction patterns ${a}_{n}^{k}\exp ({\rm{j}}{\phi }_{n}^{k})$ by Eq. (1) with different transverse distances Z_*n*_; (3) replacing the amplitude of computed patterns with the square root of recorded diffraction patterns $\sqrt{{I}_{n}}$ and retaining the computed phase ${\phi }_{n}^{k}$; (4) propagating these synthesized patterns $\sqrt{{{\rm{I}}}_{n}}\exp ({\rm{j}}{\phi }_{n}^{k})$ backward to object plane and producing *N* guesses of object ${g}_{n}^{k}(n\in [1,N])$; (5) *k*th estimation of object *G*^*k*^ is calculated by the average of *N* guessed data, i.e., ${G}^{k}=(\frac{1}{N}\sum _{n=1}^{N}|{g}_{n}^{k}|)\exp [\frac{{\rm{j}}}{N}\sum _{n=1}^{N}\arg ({g}_{n}^{k})]$ and (*k* + 1)-th input of object is assigned as ${\hat{G}}^{k+1}={G}^{k}$ for next iteration; (6) the full complex-valued image of object is obtained by running iteratively from step (2) to (5) until the reconstructed accuracy meets the required threshold.

It is not a fresh idea to introduce a feedback control to speed up the convergence for iterative phase retrieval method. As is described above, the hybrid input-output algorithm (HIO) invented by Fienup^9^ has brought feedback into the non-support region to eliminate the reluctant data so that the convergence speed is enhanced. In the HIO algorithm, assuming the input signal as ${\hat{G}}^{k}$ and the output signal as *G*^*k*^ at *k-*th iteration, The (*k* + 1)-th input is defined as

2 $${\hat{G}}^{k+1}=\{\begin{array}{ll}{G}^{k}, & \,(x,y)\in \gamma \\ {G}^{k}-\beta {\hat{G}}^{k}, & (x,y)\notin \gamma \end{array}$$

where *γ* is the support region. With the use of feedback coefficient *β*, the region of interest is enhanced and the region of redundancy is decreased. For the HIO algorithm, the tighter the support region is, the faster the convergence becomes. To get a tight support region, shrink wrap method^12,13^ is also utilized to fulfill this task. However, within a lens-based imaging system, tiny objects can be resolved and the filed of view is accordingly decreased, in which the global region is entirely interested. Thus it is impossible to seek a support region for a biological sample, such as a brain tissue or a cellular tissue. Different from these methods, our weighted feedback modality releases the requirement of the support region and is defined in the global region, which is added between steps (4) and (5) as

3 $${\tilde{g}}_{n}^{k}=(1+a+b){g}_{n}^{k}-a{\tilde{g}}_{n}^{k-1}-b{\tilde{g}}_{n}^{k-2},$$

where ${\tilde{g}}_{n}^{k}$ is the output after feedback operation for *n-*th object guess at the *k-*th iteration. Let ${\tilde{g}}_{n}^{k}={g}_{n}^{k}$ at the first two iterations, Eq. (3) will begin after the third iteration. The parameters (*a*, *b*) are feedback coefficients. If *b* = 0, the feedback is changed into the single mode. Equipped with the weighted feedback, the convergence speed of APR algorithm can be easily increased, which will be described in the following. For simplicity, we term the APR algorithm based on single and double feedback as the APRSF and the APRDF algorithm, respectively.

## Numerical fitting

**Figure 3:**
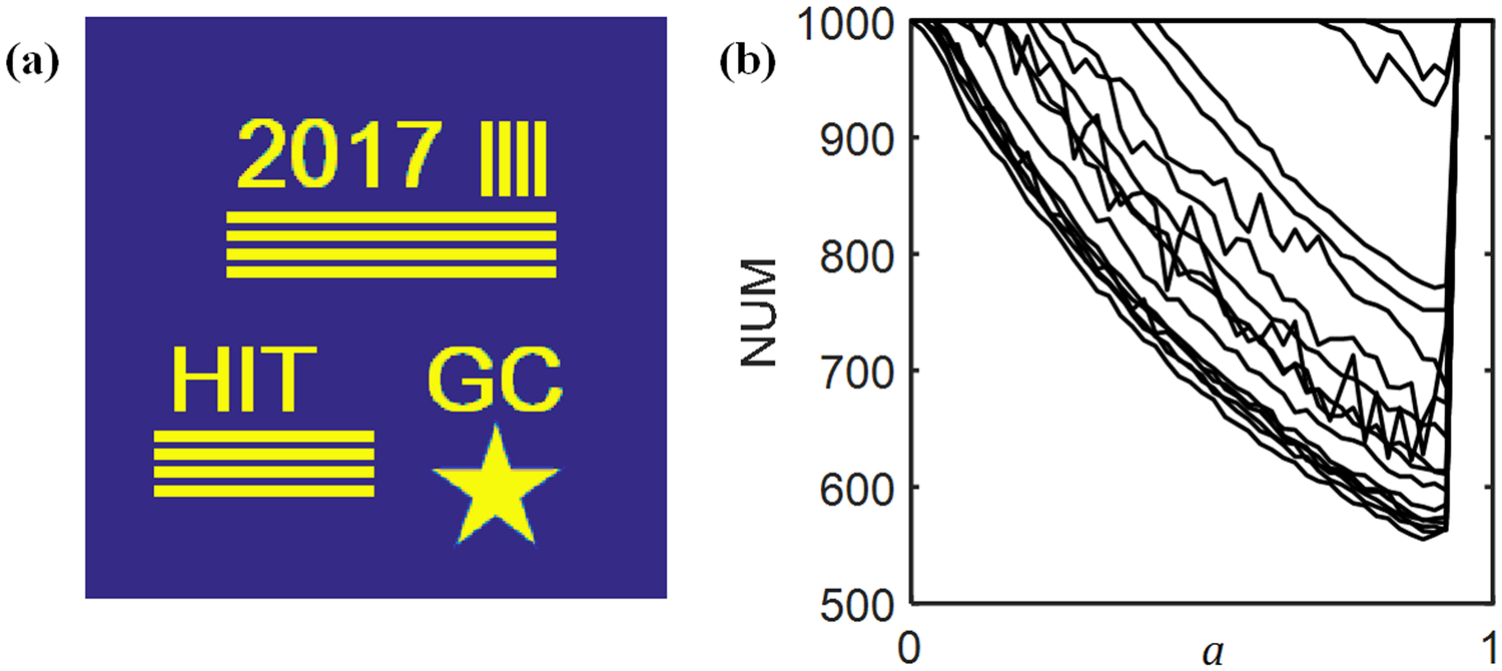
Numerical fitting for APRSF algorithm. (**a**) Ground truth image. (**b**) NUM curves with *a* ranging from 0 to 1.

We use the data-driven fitting to characterize the optimal feedback coefficients for the APRSF and APRDF algorithms. The logarithm of mean square error (LMSE) between the ground truth image and the retrieved amplitude is employed as a metric indicator for the convergence. For APR algorithm, its convergence threshold keeps around −25 orders of magnitude. If LMSE <10^−25^, the retrieved result is considered fully convergent. Hence, the convergence speed is indicated by the number of iteration (NUM) at which the recovered amplitude is less than the preassigned threshold (10^−25^). Therefore, the value of NUM is used to evaluate the convergence speed. The less NUM is, the faster the convergence speed is. The simulated parameters are listed as follows: (1) the wavelength λ is 632.8 nm; (2) the image size is 1 mm × 1 mm (256 × 256 pixels); (3) iterative number is 1000; (4) the number of recording plane *N* is 3; (5) diffractive distance includes: Z_0_ = 10 mm, *d* ∈ [15, 25] × mm, step size = 1 mm and Z_0_ = 20 mm, *d* ∈ [10,30] × mm, step size = 5 mm, for a total of 16 groups. As for single feedback, 16 groups of NUM curve are illustrated in Fig. 3(b) with the plugin of *a* (*a* ∈ [0, 1], step size = 0.02). We note that these curves have a common minimum, which is demarcated in 0.9. Thus, *a* = 0.9 is the optimal value of accelerating convergence for the APRSF algorithm.

**Figure 4:**
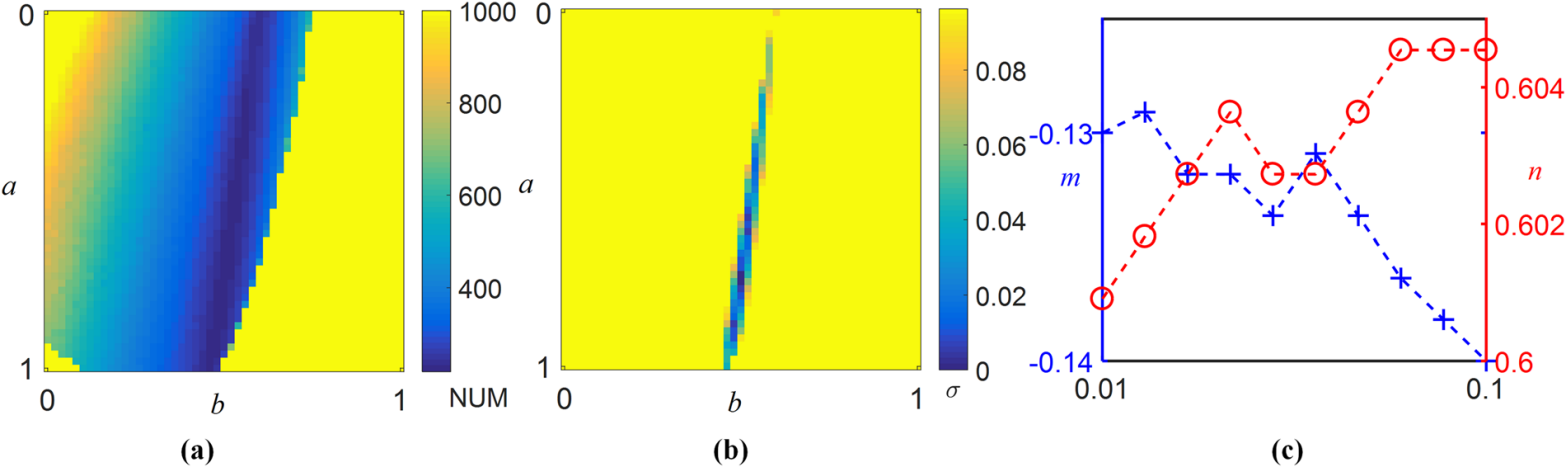
Curve fitting for APRDF algorithm. (**a**) The NUM map in Z_0_ = 10 mm, *d* = 20 mm. (**b**) The extracted NUM map of (**a**) while *σ* < 0.1. (**c**) The statistical distribution of *m* and *n* with *σ* from 0.01 to 0.1.

Curve fitting is utilized for the characterization of the APRDF algorithm. We run the APRDF algorithm with the different pairs of (*a*, *b*) (*a*, *b* ∈ [0, 1], step size = 0.02) to compute 16 NUM maps, one of which is displayed in Fig. 4(a). Especially, 16 maps like as Fig. 4(a) are calculated with a sampling size of 51 × 51, which is computation-heavy and gives a reliable guarantee for linear fitting. Here NUM map is manifested by a colorbar from yellow to deep blue. For yellow part, it represents non-convergent region under 1000 iterations. Others denote the fast-converging region. The NUM in central region is ~300, whose structure looks like an oblique line while *a* ranges from 0 to 1. Here convergence coefficient *σ* is introduced as

4 $$\sigma =\frac{{\rm{NUM}}-{{\rm{NUM}}}_{{\rm{\min }}}}{{{\rm{NUM}}}_{{\rm{\min }}}},$$

to extract the fastest region. NUM_min_ is corresponding to the minimum of each NUM map. For example, if *σ* = 0.1, the region of *σ* > 0.1 is deleted and the region of *σ* ≤ 0.1 is retained. Then the selected region is pictured in Fig. 4(b). Here the selected region is used for curve fitting and thus depicted by linear curve fitting as

5 $$b=ma+n,$$

where *m* and *n* are pending parameters. Here each pair of (Z_0_, *d*) related to one *σ* could produce a selected NUM map and then each selected map is able to be fit as a linear equation. Given a value of *σ*, we can derive *m* and *n* by $m(\sigma )=\tfrac{1}{16}\sum _{i=1}^{16}{m}_{i}(\sigma )$ and $\,n(\sigma )=\tfrac{1}{16}\sum _{i=1}^{16}{n}_{i}(\sigma )$. The variables *m*_*i*_ and *n*_*i*_ are calculated by linear fitting for a pair of (Z_0_, *d*) under a value of *σ*. Therefore, the statistical distribution of *m* and *n* is pictured in Fig. 4(c) while *σ* ranges from 0.01 to 0.1. As shown in Fig. 4(c), the parameters *m* and *n* vary with the increase of σ. Here setting *σ* from 0.01 to 0.1 could provide a reliable guarantee for linear curve fitting. Specifically, if *σ* > 0.1, the structure of the selected region is not approximated to be an oblique line. If *σ* < 0.01, it is impossible to ensure that all selected maps have two or more sampling data to use linear fitting. Here *m* and *n* are parameterized by keeping all values of statistical distribution to the second decimal place. With this process, the parameter *n* is easy to be truncated as 0.60 but *m* is approximated to −0.13 and −0.14. Note that the statistical distribution of *m* dramatically decreases if *σ* > 0.6, the values (*σ* > 0.6) are regarded as redundant and unstable data for curve fitting. Thus *m* is roughly assessed at −0.13. Accordingly, Eq. (5) is parameterized as

6 $$b=-0.13a+\mathrm{0.6.}$$

### Simulation

**Figure 5:**
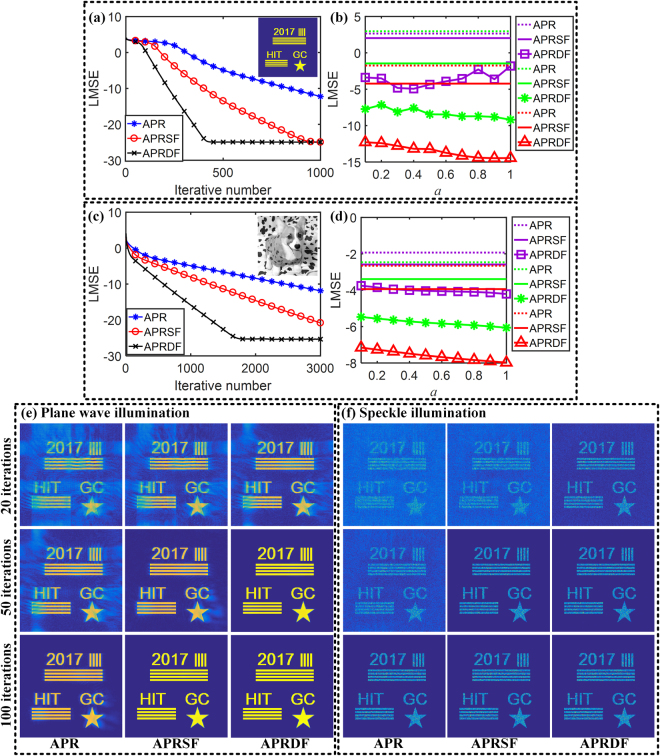
Numerical simulation for APR, APRSF and APRDF algorithm. (**a**) and (**c**) are LMSE curves retrieved from binary and grey-scale object for APR, APRSF (*a* = 0.9) and APRDF algorithm (*a* = 0.7, *b* = 0.5) in Z_0_ = 10 mm, *d* = 12 mm, *N* = 3, iterative number = 1000. (**b**) and (**d**) are LMSE distributions retrieved from binary and grey-scale object with the wavelength of 432 nm (purple curve), 532 nm (green curve) and 632 nm (red curve) in Z_0_ = 20 mm, *d* = 2 mm, *N* = 4. (**e**) and (**f**) are reconstructed images after 20, 50 and 100 iterations in Z_0_ = 10 mm, *d* = 1 mm, *N* = 11 under plane wave and speckle illumination.

Numerical simulation is given to exhibit the performance of our weighted feedback scheme. Let *a* = 0.9 for the APRSF and *a* = 0.7, *b* = 0.5 for the APRDF, the LMSE curves of three algorithms (Z_0_ = 10 mm, *d* = 12 mm, *N* = 3, 1000 iterations) are illustrated in Fig. 5(a) for binary objects. The APR, APRSF and APRDF spend 56s, 60s and 61s for computation in total (Intel i5 CPU, no GPU), as shown in Fig. 5(a). But the APRDF reaches the convergence with only ~400 iterations. Accordingly, the convergent time of the APRDF is merely ~30s. Working the Eq. (6) with a series of *a*, the LMSE distribution under different wavelengths (432 nm, 532 nm and 632 nm) is shown in Fig. 5(b), where its setting parameters are listed as: Z_0_ = 20 mm, *d* = 2 mm and *N* = 4, iterative number = 1000.

In Fig. 5(b), *a* = 0.9 is initialized for the APRSF. As different values of *a* (0.1 ≤ *a* ≤ 1) are put into Eq. (6), the reconstructed results from pairs of (*a*, *b*) are plotted for theAPRDF. For each wavelength, the reconstructed accuracy of the APRDF holds the first place even if the feedback coefficient *a* changes from 0.1 to 1, which proves the effectiveness and stability of Eq. (6). Similar to Fig. 5(a,b), the LMSE curves and distribution of grey-scale object are shown in Fig. 5(c) and (d). Especially, all simulation parameters are the same as in Fig. 5(a) and (b), except that the computation is run with 3000 iterations. We note that the single and double feedback both speed up the convergence for the complicated sample. In the iterative phase retrieval method, more recording planes could heavily reduce the number of iteration. These results have shown that weighted feedback is feasible for the multi-distance phase retrieval even with more recording planes. Thus the simulations of *N* = 11 are displayed in Fig. 5(e) for a plane wave illumination and in Fig. 5(f) for a speckle illumination. As is shown in Fig. 5(e), the APRDF algorithm retrieves the object only after 50 iterations. On the contrary, it is still non-convergent after 100 iterations for the APR algorithm.

It has been proved in refs^29,30^ that a random phase illumination could lead to a unique and stable reconstruction to solve the problem of ambiguity, trivial and nontrivial for iterative phase retrieval. As its typical implementation, the speckle illumination caused by a diffuser has been introduced into digital holography for a unique and fast solution in refs^22,27,28,31^. Here the weighted feedback can also be used in the speckle illumination. To simulate the speckle field, a phase mask with a range of [0, 1.2π] is placed upstream 10 mm from the object. It clearly shows that the APRSF and the APRDF could reconstruct a full target only after 50 iterations, while the APR needs 100 iterations. The results are shown in Fig. 5(f). Obviously, the quality of recovered images in Fig. 5(f) is impaired due to the speckle illumination although the structure of object is resolved. We believe that this problem deserves to be perfected in the future. In this section, it is proved that weighted feedback is capable of getting a fast reconstruction for different samples.

## Experimental Results

**Figure 6:**
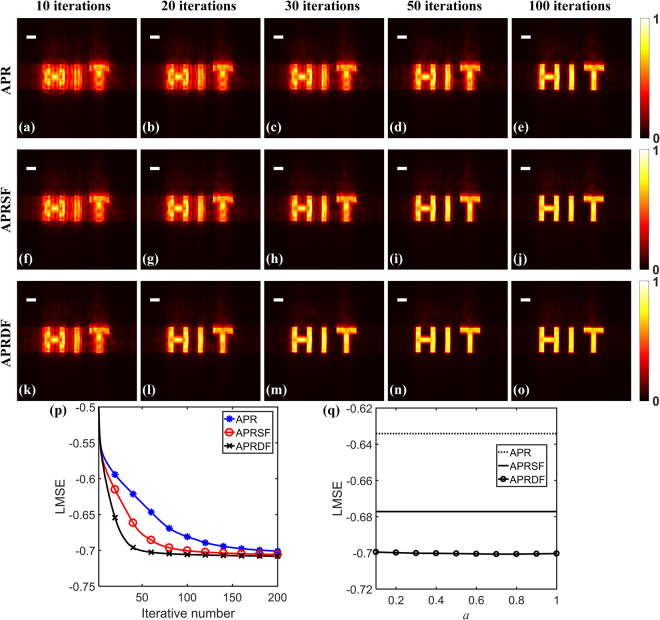
The reconstructed images under coherent illumination. (**a**–**e**), (**f**–**j**) and (**k**–**o**) are retrieved by APR, APRSF and APRDF algorithm with 10, 20, 30, 50 and 100 iterations. (**p**) The convergence curve for three algorithms. (**q**) LMSE distribution after 50 iterations. The white bar corresponds to 150 μm.

**Figure 7:**
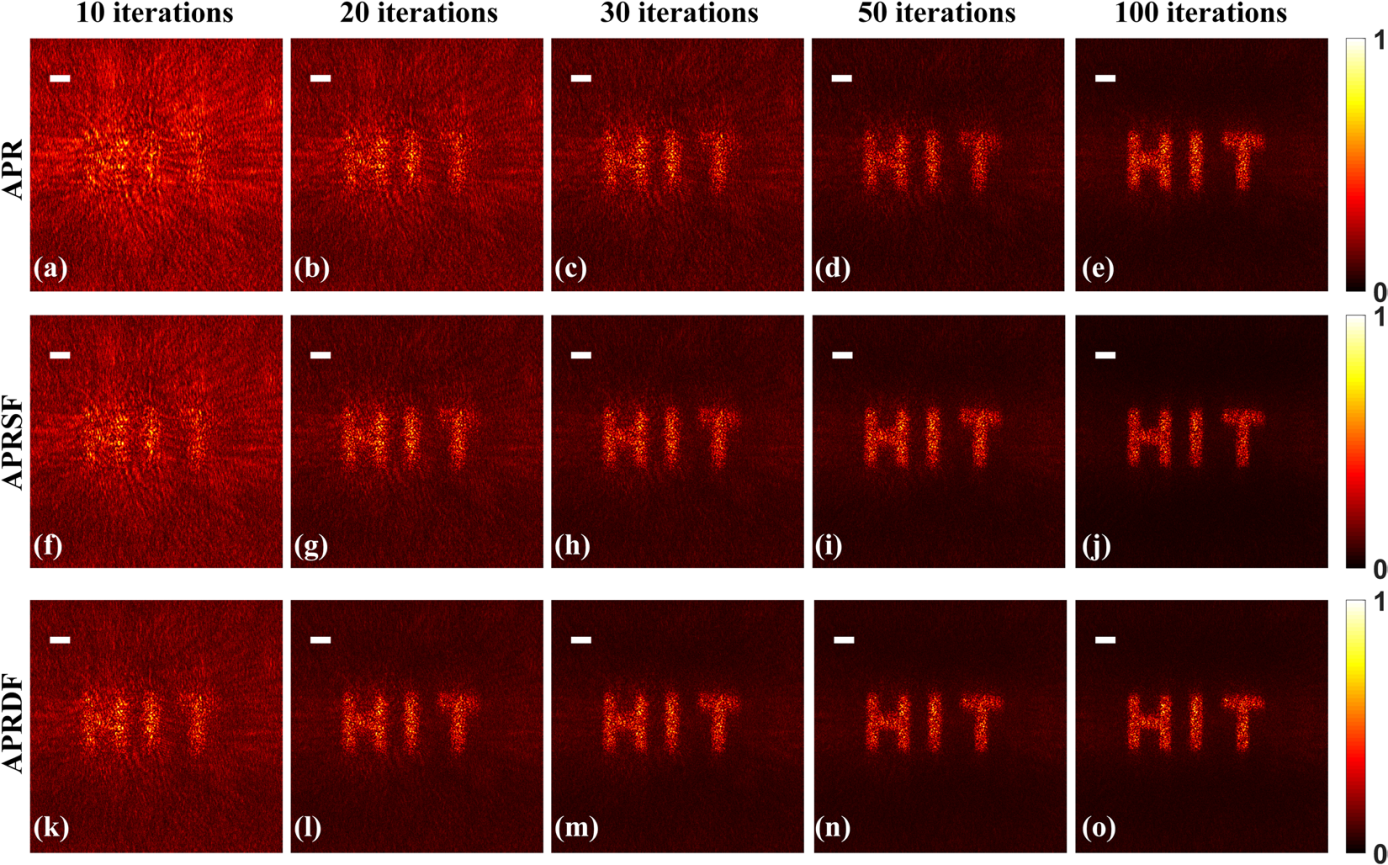
The reconstructed images under speckle illumination. (**a**–**e**), (**f**–**j**) and (**k**–**o**) are retrieved by APR, APRSF and APRDF algorithm with 10, 20, 30, 50 and 100 iterations. The white bar corresponds to 150 μm.

We conduct imaging experiments in the lens-less system and the typical wide-field microscope. The fundamental set-up of the lens-less imaging is displayed in Fig. 1. A diverging spherical wave is used to illuminate a collimating lens to generate parallel light. Subsequently, the shaped plane wave through an aperture illuminates the sample to produce a dataset of diffraction patterns with the initial distance Z_0_ and the equally-spaced interval *d*. The receiving device is a CCD camera (3.1 μm, Point Gray), which is mounted on a precision linear stage (M-403, resolution 0.012 μm, Physik Instrumente Inc.) to implement transverse movement. A fiber laser with the wavelength of 532 nm is used for the coherent illumination and a target of ‘HIT’ etched on a glass is regarded as the sample. Here *a* = 0.7 and *b* = 0.5 deriving from Eq. (6) are fed for the APRDF algorithm and *a* = 0.9 for the APRSF algorithm. The central 600 × 600 pixels of CCD is chosen as the imaging region for the recorded patterns. The other experimental parameters are listed as: Z_0_ = 21 mm, *d* = 1 mm, *N* = 11, iterative number is selected as 10, 20, 30, 50 and 100. The relevant results are presented in Fig. 6 for the coherent illumination and in Fig. 7 for the speckle illumination. In Fig. 6, with the increase of iterations, the structure of reconstructed image is visually resolved for three algorithms. From Fig. 6(e),(i) and (m), we can see that the sample is fully reconstructed after 100, 50 and 30 iterations by the APR, APRSF and APRDF algorithm, which correspondingly takes 151s, 86s, 50s for computation. Thus single and double feedback could speed up the convergence with a factor of two and three. To quantitative show this improvement, the LMSE curves are plotted in Fig. 6(p), where the acceleration effect is remarkable for the weighted feedback. Especially, in experiments, the values of LMSE are calculated at the recording plane, i.e., using the square root of recorded intensity as the ground truth. Plugging different values of *a* (0.1 ≤ *a* ≤ 1) into Eq. (6), the LMSE distribution is pictured in Fig. 6(q) with different pairs (*a*, *b*) after 50 iterations. The results show that, in Fig. 6(q), the APRDF algorithm still holds the stable and fast-converging reconstruction even if the feedback coefficients are different, which actually agree with the simulations.

The imaging results with the speckle illumination are shown in Fig. 7. The experimental parameters as same as in Fig. 6, except that a ground glass diffuser (DG10-120-MD, Thorlabs, 120 grit) is inserted between collimated lens and sample, to generate speckle field. Accordingly, the retrieved images of three algorithms are displayed in Fig. 7. Similarly, the APRDF, APRSF and APR fulfill the image reconstruction with the iterations of 30, 50 and 100, where the computational time is 48s, 77s and 141s. The results again show that the weighted feedback has the ability of speeding up the convergence for the multi-distance phase retrieval.

**Figure 8:**
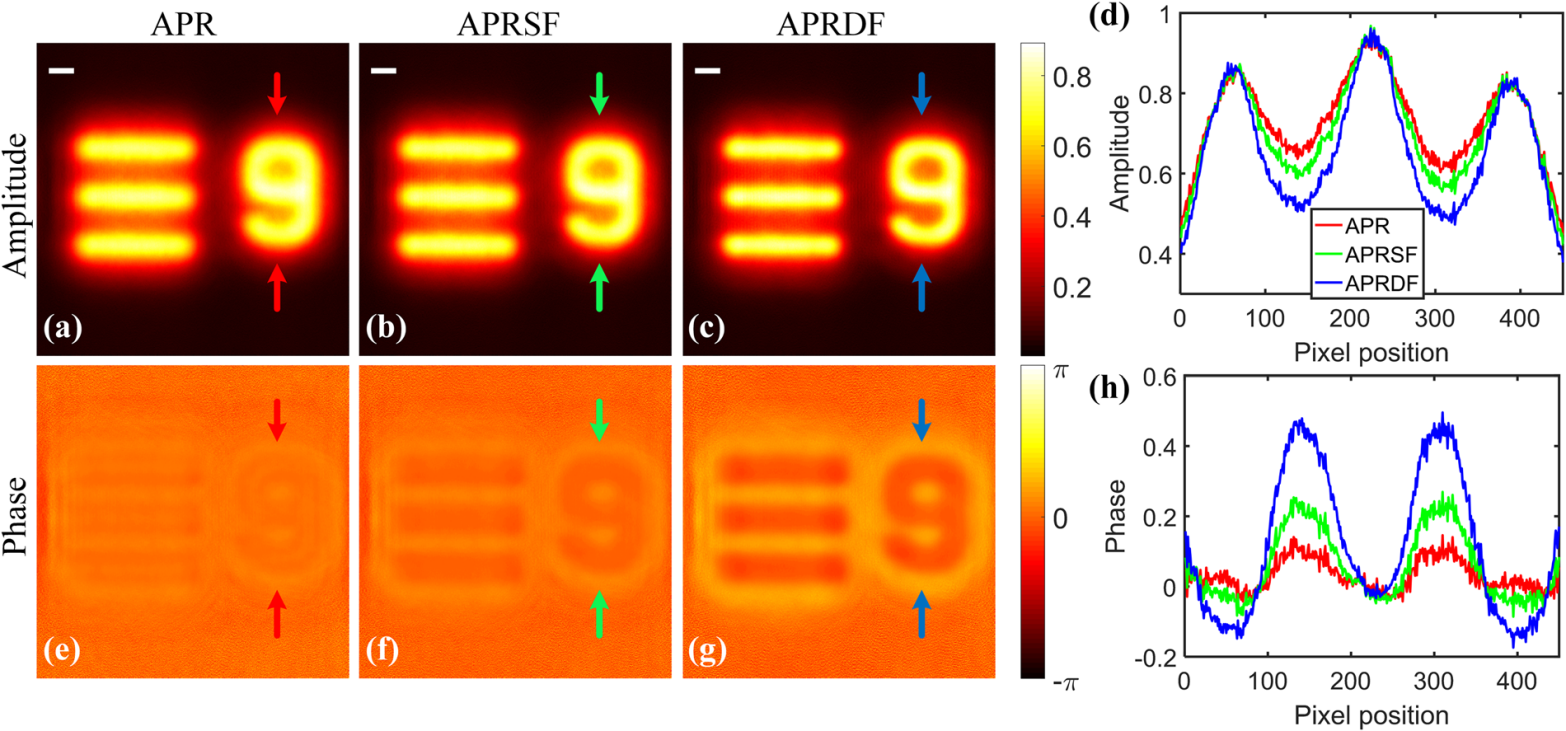
The reconstructed images under partially coherent illumination. (**a**–**c**) and (**e**–**g**) are retrieved amplitudes and phases by APR, APRSF and APRDF algorithm. (**d**) and (**h**) are the plot lines between red, green and blue arrows from (**a**–**c**) and (**e**–**g**). The white bar corresponds to 300 μm.

Additionally, the weighted feedback is helpful for the enhancement of imaging contrast. Replacing the fiber laser with a LED, the reconstruction under partially coherent illumination is shown in Fig. 8. We use a programmable LED matrix (Adafruit 607) to illuminate condenser lens (f = 80 mm) to produce a parallel beam (532 nm) and other devices are unchanged. Practically, different LEDs are switched on to adjust optical system so as to eliminate the impact of off-axis. Once on-axis is calibrated, the only one LED corresponding to the used position is applied for illumination.

An inverted symbol ‘6’ and a cross stripe of 1951 USAF resolution chart (R3L3S1N, Thorlabs) are served as the sample. The imaging size of the recorded patterns is 1200 × 1200 pixels and other experimental parameters are listed as follows: Z_0_ = 74 mm, *d* = 3 mm, *N* = 4, and iterative number is 200. The APR algorithm is invented in the case of coherent illumination. It is no doubt that its reconstructed amplitude and phase are degraded by partially coherence in Fig. 8(a) and (e). As we expect, the degradation caused by partial coherence can be eliminated by our augmented approaches. Especially for the APRDF algorithm, it significantly enhances the contrast of amplitude and phase in Fig. 8(c) and (g). Furthermore, the APRDF acquires an excellent visual effect on phase contrast and two others are incompetent. Capturing data between two red, green and blue arrows on recovered images, the slicing outlines are drawn in Fig. 8(d) and (h), in which single and double feedback both function well to enhance contrast of amplitude and phase. This experiment proves that the APRDF algorithm not only provides a fast-converging strategy but also achieves a full reconstruction of object function under partially coherent illumination.

**Figure 9:**
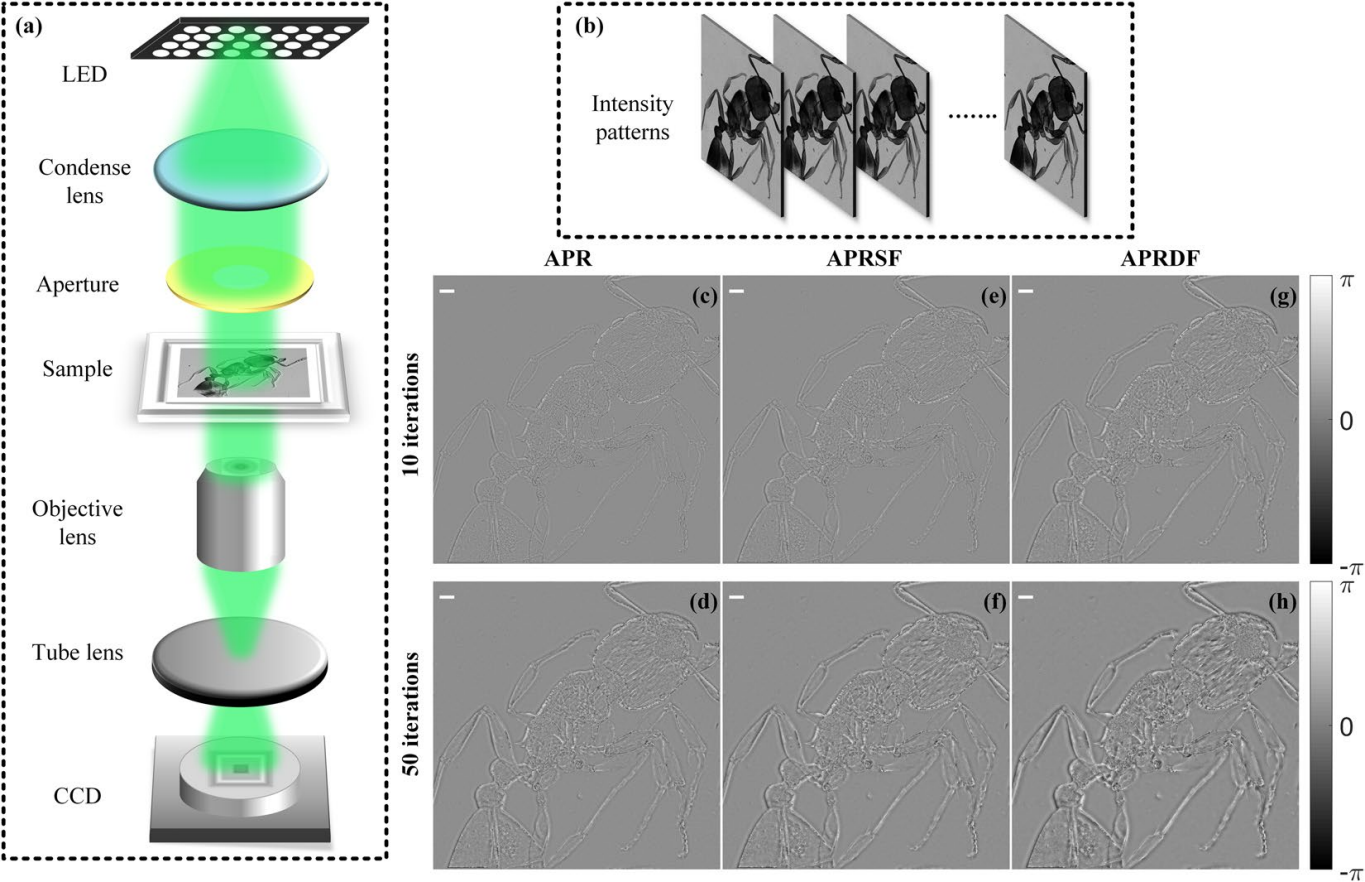
The reconstruction of ant specimen in wide-filed microscope. (**a**) The experimental schematic. (**b**) The recorded dataset of intensity patterns. (**c**,**d**) are reconstructed phases for APR algorithm, (**e**,**f**) for APRSF algorithm and (**g**,**h**) for APRDF algorithm after 10 and 50 iterations. The white bar corresponds to 120 μm.

To further prove the capability of our method, we apply these methods in the wide-field microscope and the corresponding experimental schematic is shown in Fig. 9(a). The 4f arrangement is composed of a tube lens (f = 200, ITL200, Thorlabs) and an objective lens (4×/0.13, Nikon). A beam of plane wave shaped from condense lens (f = 80 mm) and adjustable aperture illuminates ant specimen to produce a set of intensity patterns. The receiving detector is CCD camera (3.1 μm, Point Gray). The dataset of defocused intensity patterns is recorded by mounting CCD camera on a linear stage (M-403, resolution 0.012 μm, Physik Instrumente Inc). 21 intensity images nearby in-focused plane are sequentially received and the focusing method in ref.^32^ is utilized to acquire in-focused image. After this preparation, one in-focused and ten defocused intensity patterns are recorded with an equal interval of 0.2 mm, which are displayed in Fig. 9(b). With the use of the APR, APRSF and APRDF algorithms, the phases of an ant specimen are retrieved in Fig. 9(c–h) after 10 and 50 iterations. In this case, an in-focused image is regarded as object constraint, i.e., replacing the amplitude of object estimation derived from the average of *N* guesses with the square root of in-focused intensity pattern. Thus the output image with the APR, APRSF and APRDF algorithms is the phase of the ant specimen. As shown in Fig. 9(c),(e) and (g), the structure of ant retrieved by the APRDF is dominant to be resolved by contrast with APR’s and APRSF’s one. Also, as the weighted modality changes from single to double mode, the phase contrast of ant specimen is strengthened. With the increase of iterations, the APR enables the ant specimen more clearly in Fig. 9(d). But the APRDF sharpens the edge of ant specimen and achieves the imaging contrast enhancement at the highest level in Fig. 9(d),(f) and (h). These results prove that weighted feedback facilitates the enhancement of the imaging contrast for iterative phase retrieval.

## Conclusions

We developed a fast-converging modality, weighted feedback, to accelerate the convergence for multi-distance phase retrieval. Based on the APR algorithm, two augmented approaches, namely, the APRSF and the APRDF, are proved to speed up the convergence in simulation, where the optimized feedback coefficients are parameterized by numerical fitting. In experiments, two algorithms, the APRSF and the APRDF, have been demonstrated to speed up the convergence with a factor of two and three under coherent and speckle illuminations. Another advantage of the weighted feedback embodies in the enhancement of imaging contrast. Furthermore, the APRDF algorithm has the ability of achieving a full high-contrast reconstruction of complex amplitude for both binary object and biological specimen, which extends multiple distance phase retrieval to the application of partially coherent illumination. Therefore, the weighted feedback scheme will provide a fast-converging and high-contrast imaging modality for iterative phase retrieval.

The limitations of the weighted feedback APR lies in two aspects. First, the weighted feedback not only enhances the imaging contrast for the region of interest but also magnifies the background noise. Accordingly, this modality deserves to be improved by combing denoising method. Second, the feedback modality in our work merely reaches to a double mode. More investigations of multi-feedback modality will be a prospective challenge in the field of iterative phase retrieval.
